# Supplementary material for: Poor adult tuberculosis treatment outcome and associated factors in Gibe Woreda, Southern Ethiopia: An institution-based cross-sectional study
Source: PLOS Glob Public Health. 2022 Mar 10;2(3):e0000161. doi: 10.1371/journal.pgph.0000161 (PMC10021194; doi:10.1371/journal.pgph.0000161)
Supplement: Attachment — Submitted filename: Responce to Reviewers.docx [file pgph.0000161.s002.docx]

**November 17, 2021**

Rebuttal letter for paper PGPH-D-21-00626 “Poor adult tuberculosis treatment outcome and associated factors in Gibe Woreda, Southern Ethiopia: an institution-based cross-sectional study”
PLOS Global Public Health

**Dear the editor and reviewers,**

The authors are very thankful of the academic editor’s and reviewers’ invaluable contributions and the time they invested in reviewing this manuscript and for the subsequent improvement that followed. We considered all their comments and suggestions and corrected the manuscript accordingly. The changes made are highlighted in track changes in the revised manuscript. Due to the changes made, however, the line numbers in previous document may not be the same as in the revised document. We provided the point-point response to concerns raised by both the editor and reviewers. We believe that the changes in the manuscript that followed in addressing the editor’s and reviewers’ concerns have considerably improved our manuscript, and we hope that it now stands out for publication.

**Point-by-point responses**

**Academic editor**

- If you are reporting a retrospective study of medical records or archived samples, please ensure that you have discussed in the ethics statement whether all data were fully anonymized before you accessed them and/or whether the IRB or ethics committee waived the requirement for informed consent. If patients provided informed written consent to have data from their medical records used in research, please include this information.
  - *Thank you for this comment. We have now included a statement that describes the anonymous collection of data. However, instead of obtaining patient consent, the ethics review committee approved consent to access data to be sought from the respective health facility heads as it could be difficult to reach to patients.*
- In the online submission form, you indicated that "Data are available upon request from the corresponding author." All PLOS journals now require all data underlying the findings described in their manuscript to be freely available to other researchers, either 1. In a public repository, 2. Within the manuscript itself, or 3. Uploaded as supplementary information. This policy applies to all data except where public deposition would breach compliance with the protocol approved by your research ethics board. If your data cannot be made publicly available for ethical or legal reasons (e.g., public availability would compromise patient privacy), please explain your reasons by return email and your exemption request will be escalated to the editor for approval. Your exemption request will be handled independently and will not hold up the peer review process, but will need to be resolved should your manuscript be accepted for publication. One of the Editorial team will then be in touch if there are any issues.
  - *Thank you for this suggestion. We have now provided the data as a supplementary/supporting information file in the revised submission. We also corrected the previous statement in the revised manuscript.*
- 3. Please amend your detailed Financial Disclosure statement. This is published with the article, therefore should be completed in full sentences and contain the exact wording you wish to be published. i) State the initials, alongside each funding source, of each author to receive each grant. ii). State what role the funders took in the study. If the funders had no role in your study, please state: “The funders had no role in study design, data collection and analysis, decision to publish, or preparation of the manuscript.”
  - *Thank you for this suggestion. We have now corrected the financial disclosure as suggested in the revised document and also specified the funder’s role.*
- In your Financial Disclosure, you indicated "Funding was obtained from Haramaya University, College of health and medical sciences.", but in the Funding Information you indicated that no funding was received. Please revise the Funding Information field to reflect funding received.
  - *Thank you very much for this helpful suggestion. We have corrected the funding information in this revised submission.*

**Reviewer #1**

- In this manuscript, the authors use data from clinical charts of adult TB patients attending four centres in Gibe Woreda, Ethiopia, to describe treatment outcomes and assess factors associated with poor outcomes. Amongst 400 patients included in the analysis, 58 (14.5%) had poor outcomes, and the multivariable analysis suggested that such outcomes were more common amongst men, the oldest age group, those travelling further to the health facilities, and those without family support. The results seem plausible, but I am concerned that the data available may not be able to support the multivariable analysis.
  - *Thank you for the kind words. We have tried to address the important concerns you have raised in the revised manuscript.*
- This is a sub-analysis of data collected for another study, so the sample size is based on the requirements for that study (n = 400). It is not clear how well this available sample size can address the objectives of this analysis.
  - *Thank you for this very important concern. This current study used the same sample size as that of the previous study where only two transferred out cases were excluded in this study resulting in the final sample size of 400. As a rule of thumb (as you well know), for a multivariable regression analysis, there should be at least 30 observations available per an independent variable. Accordingly, in our study, only ten variables were included in the analysis, and the available sample size is well beyond the minimum required observation.*
- There are very few patients with poor outcomes in some groups, leading to very wide confidence intervals, particularly in the multivariable analysis. I am not sure how far this can be mitigated, but one or more of the following might help:
- - For some variables, it might help somewhat if a different group is taken as the baseline (for example, for the age variable, taking the >=45 years group as the baseline as this has the largest number of participants).
  - *Thank you very much for the very helpful suggestion. Considering your suggestion and that of the third reviewer, we have now entered age as a continuous variable in the multivariable analysis to avoid scattering of observations across categories.*
- - It may also be useful to reduce the number of categories for some variables, but only if it is meaningful to do so. For example, there are small numbers in several of the occupation categories and perhaps some of the groups could be combined. However, this might produce heterogeneous groups and the authors might not think this is appropriate.
  - *Thank you for this comment. We have now reduced the number of categories for occupation to three and run the model and we noted some improvement in the confidence intervals for effect estimates.*
- - The number of variables in the multivariable model could be reduced. Currently there are at least 10 variables included (some with multiple categories), which is a lot given that there are only 52 events (patients with poor outcomes). For example, is it possible to select a few variables of primary interest (based on prior work and / or plausibility) rather than selecting variables for inclusion in the model based on p values in the univariable analysis?
  - *Thank you very much for the comment. In the revised manuscript, as most of the patients had disclosed their status and also received family support (78.8%), we removed “disclosure of TB status” and run the model which provided us with a gain in the precision of effect size estimates as rightly suggested.*
- Also regarding the analysis, did the authors consider adjusting for clustering by site, e.g. using multilevel logistic regression?
  - *Thank you very much for this interesting comment. However, the authors did not design the study in that way, and hence we did not collect data that will enable us to look into the clustering effect. We accept the comment, and include this as a limitation.*
- The text lists 12 variables which were included in the multivariable model, but two of these (nutritional support and type of TB) do not appear in Table 3.
  - *Thank you for the comment. It was an editorial error, and we have corrected it in the revised manuscript.*
- The sample size section refers to 402 patients being included in the original study, although 400 are included in this analysis. What were the reasons for excluding the other two patients from this analysis?
  - *Thank you for the concern on this. The two patients excluded in the current study as they were transferred out and their treatment outcomes were not known. We included the detail on this in the revised document.*
- Some of the categories in Table 1 need to be defined. For example:

- Are reactive and non-reactive (HIV status) equivalent to positive and negative?

- *Thank you for the comment. We considered the terms reactive and non-reactive as an equivalent to positive versus negative for HIV status. However, to avoid ambiguity, we used positive versus negative when referring to HIV status in the revised manuscript.*

- What are the definitions of severe, moderate and normal nutritional status?

- *Thank you for the comment. We acknowledged the comment and incorporated definition for nutritional status as follows using body mass index (BMI): severely underweight (BMI < 16.5kg/m2), underweight (BMI under 18.5 kg/m2) , normal weight( BMI ≥ 18.5 to 24.9 kg/m2) , overweight (BMI ≥25 to 29.9 kg/m2), and obesity – BMI ≥ 30 kg/m2(The reference is cited in the revised manuscript). The nutritional status classification in the previous submission was corrected as given above in this revised submission.*

- What is meant by having a contact person?

- *Thank you for the comment. This was also a typo error and we corrected it in the revised submission as “having history of contact with TB patient” which was defined as sharing the same enclosed living space/house for one or more nights or for frequent or extended periods with the index case during the 3 months before the diagnosis of TB.*

- How were good and fair medication adherence defined?

- *Thank you for the comment asking for clarification on this. In the revised document, we redefined adherence status as adherent versus non-adherent as this classification was widely used. A TB patient either on the intensive or continuation phase under a new or retreatment regimen who missed ≥ 10% of the total prescribed TB medication dose was considered as non-adherent, and otherwise adherent. This detailed addition was included in the revised manuscript.*

- What was considered a side effect?

- *Thank you for the concern raised on this. In this study, side effect was defined as any adverse reaction that resulted in discontinuation of anti TB drugs, and/or directly resulted in hospitalization. We have included this elaboration in the revised manuscript.*

- Does disclosure status refer to HIV disclosure? The text refers to HIV disclosure but the tables include data on disclosure status for all participants, not just those with HIV. Please clarify and define.

- *Thank you for the comment. The disclosure variable in this study did not refer to HIV but to TB disclosure. However, we included a qualifying text on this in the revised manuscript.*
- It would be helpful for Tables 1 and 2 to stratify by good / poor outcome status
  - *Thank you. In Tables 1 and 2, the outcomes are now stratified by poor and good TB treatment in the revised manuscript.*
- **Minor comments**
  - Line 69: the word “defaulter” is used – this is no longer a preferred term, I suggest replacing with lost to follow-up.
    - *Thank you for the comment. We acknowledged the comment and corrected the term by lost to follow-up in the revised manuscript.*
  - Lines 196-199: please give the numbers (numerator and denominator) with poor outcomes each year as well as the percentages.
    - *Thank you for the comment. Percentages of poor treatment outcome per each year considered with corresponding numerator and denominator was given in the revised manuscript.*
  - Line 155: I think there is a typo – variable inflation factor should be variance inflation factor?
    - *Thank you for the comment. The indicated typo error was corrected in the revised submission as ‘variance inflation factor’.*
  - Line 201-202: “Among adult TB patients who had no family support (28), 23 (5.75 %) experienced poor TB treatment outcomes.” Based on Table 2, I think this might need to be rephrased – are you saying that of 51 patients who had no family support, 23 experience poor TB treatment outcomes? I am not sure what the 28 and 5.75% refer to.
    - *Thank you for the comment. The statement made was to communicate that among adult TB patients who had no family support (51) (28), 23 (5.75%) experienced poor TB treatment outcome. This clarification was now added in the revised manuscript.*
  - I think the columns for occupation status in Table 3 may be the wrong way around (total of 342 in the poor outcome column and 58 in the successful column).
    - *Thank you for the comment. Of course, it was wrong, and we have corrected it in the revised manuscript.*

**Reviewer** #**2**

- This is one of the important article as DRTB present major abstacles in the control of TB and it is very important to find out factors responsible for poor outcomes of DSTB as succesful outcomes of TB treatment may help in the cutt off the chain to convert to drug resistant TB. Good work
- *Thank you for the kind words implicating the public health importance of our work in prevention of DRTB.*

**Reviewer** #**3**

**Major comments**

- The sample size determination and sampling procedure section starting in line 108 is confusing, particularly considering lines 104-105. In line 105 it indicates that all adults with TB were considered, but in line 114 it states that a sampling technique was considered. What was ultimately done?
  - *Thank you for the comment. In the ‘original study’, the minimum sample size estimated was 417, and 402 were included in the final analysis. As previously suggested, we used the sample size estimated for the previous(original study) study. As the previous study used a survival analysis, the follow-up time of those who developed the outcome interest and those who did not are considered, and hence the two excluded cases in the current study were maintained in the previous study as the model used for analysis was different. Regarding sampling technique, a simple random sampling was used to select the minimum sample from 1211 eligible TB patients who received care in the selected health facilities in the period specified.*
- Was this a convenience sample with a target of 402, or were all adult TB patients included (except for those who transferred out)?
  - *Thank you for the comment. The sample was not drawn based on convenience rather using a simple random sampling from list of 1211 adult TB patients who received first line TB-treatment between June 2016 and June 2019.*
- Since the Results report 400 patients included in the analysis, it seems that this did not include all eligible patients. On what basis were the 400 patients chosen?
  - *Thank you for the comment. As described in the response given above, for the two patients excluded in this study, we couldn’t determine their treatment outcome as they were transferred out cases. Therefore, the remaining 400 TB patients with a defined treatment outcome are included in the current analysis.*
- The details reported in lines 109-113 lack context, and what does “exposed to an unexposed group” mean? This entire section needs to be revised for clarity
  - *Thank you for the comment. In the original study, ‘exposed’ refers to patients who travelled 10 km and above to receive TB treatment, whereas ‘unexposed’ were patients travelled less than 10 km to receive TB treatment. This clarification has now been added in revised document.*
- Results: Out of how many eligible TB patients were the 400 chosen? Are there any indications how these differ from those who were not chosen?
  - *Thank you for the concern. The sample studied (400) was selected from a total of 1211 TB patients in all the facilities involved. Sample from each health facility was drawn proportionally to the size of patients who received TB care using a simple random sampling. As the sample was selected randomly from the eligible study population per facility, we assume those participated in the study and those not included were similar in terms of important variables. Furthermore, the simple random sampling technique was also employed assuming that they were homogenous with respect of the characteristics studied.*
- Results: Why was age categorized with the highest category as 45+ years? This group had the highest frequency, making one wonder what the age distribution is above 45 years. Why not use age as a continuous variable in the analyses (could assess for linearity, use restricted cubic splines, etc.)? If age is kept in categories, I would strongly consider adding age categories with older ages. This would be helpful to interpret the Age results in the regression models. Similarly, why were body weight and distance travelled categorized, and on what basis were the cut-offs used?
  - *Thank you very much for the comments. As suggested, we included the variables ‘age’ as a continuous variable in the revised manuscript. However, we couldn’t use distance travelled in a continuous form as the data for this variable was originally collected categorically with estimated distance based on where the patient was coming from (based on his/her usual residence). The cut-off value was used based on the practice in previous researches.*
  - *In the descriptive analysis, we have re-categorized age to correct the higher frequency in the category of 45+ years to include other age groups.*
  - *Regarding the body weight, we used previous study as a base which used 35 Kg as a cut-off point in the cohort of TB patients on DOTS in Addis Ababa, Ethiopia (doi:10.1186/1471-2334-11-127).*
  - *Regarding the distance travelled, we used previous study done in Ethiopia, in Sheka Zone by Shaweno T et al on the LTFU of TB patients, which used 10 Km as a cut-off value for the distance travelled to receive TB treatment from TB treatment centre (*[*https://doi.org/10.1186/s41182-020-00198-8*](https://doi.org/10.1186/s41182-020-00198-8)*).*
- Table 2: Some of the variables require more details (in footnote, or text, or supplementary material).
- - What defines the categories of functional status variables, malnutrition categories?
  - *Thank you for the comment. The functional status was measured according to the WHO patient monitoring guidelines and details are now added as a footnote in Table 2 in the revised manuscript: working refers to, “able to perform usual work in or out of the house…;” ambulatory refers to, “able to perform activities of daily living but not able to work or play;” and bedridden refers to, “not able to perform activities of daily living.” (https://bmcpublichealth.biomedcentral.com/articles/10.1186/s12889-019-7980-x).*
- - What does “Having contact person” mean?
  - *Thank you for the comment. This was a typo error, and it was rather to refer to “having history of contact with TB patient”. This has now been modified in the revised manuscript.*
- - Do the smoking and drinking alcohol categories mean current? Ever? How much tobacco or alcohol use defines smoking or drinking? Any? A certain quantity per time?
  - *Thank you for the comment. The smoking and drinking alcohol categories are to mean current when the patient data were recorded, but we did not measure the amount of cigarette smoked and alcohol intake because it was secondary data from the record. This can be taken as a limitation of secondary data and mentioned so in the limitation section in the revised manuscript.*
- - How is medication adherence defined? Is it self-report, directly-observed therapy / medication log? Pill count? What threshold defines adherence? Is it all medications, some medications? Is it adherence to anti-TB drugs? Some of them? All of them?
  - *Thank you very much for the interesting concern. The anti-TB treatment was given in DOTS in the facilities studied. Accordingly, adherence was measured and dichotomized as adherent and non-adherent based on the percentage of missed drugs. A TB patient belonging to either intensive or continuation phase under a new or retreatment regimen who missed ≥ 10% of the total prescribed TB medication dose was considered as non-adherent, and adherent otherwise. This definition was corrected in the revised manuscript.*
- - What constitutes a side effect?
  - *Thank you for the comment. In this study, a major side effect was defined as any adverse reaction that resulted in discontinuation of anti TB drugs, and/or directly resulted in hospitalization, and a qualifying statement has now been added in the revised manuscript.*
- - What defines family support?
  - *Thank for this important question. Family support could better be understood from the perspective of the patient as it may take ranges of perceived support including reminding medication time, assisting with patient needs at home or outside, accompanying patient or covering the patient’s travel cost to health facility, and also it could be in terms of psychological support.*
  - *In the current study, we defined family support based on patient reports whether they received social support from families related to TB care (*[*https://doi.org/10.3390/ijerph17155626*](https://doi.org/10.3390/ijerph17155626)*).*
- - What defines nutritional support?
  - *Thank you for this interesting question. Nutrition support was guided by the cut-off points for body mass index (BMI) indicated in the National TB Guidelines. It is recommended for adults identified with severe acute malnutrition (SAM) (BMI<16 Kg/m2) and moderate acute malnutrition (MAM) (16 Kg/m2≥BMI<17 Kg/m2) and had TB/HIV co-infections on admission. Plumpy nut (an energy dense fortified therapeutic food designed for treatment of SAM) or plumpy sup (an energy dense fortified therapeutic food designed for treatment of MAM) was provided to those eligible as a nutritional support. Based on this recommendations, and the TB treatment guidelines of Ethiopia, those patients who received nutritional support was categorized as “yes”, and those who did not receive were categorized as “no”.*
- It seems unlikely that there was 0% unsuccessful treatment outcome in 2016. How were the patients assigned to treatment year? Year of treatment start? Year of recorded TB treatment outcome? How many patients had treatment outcomes in 2016? Could it be that since the study period was June 2016 that very few people included in the study had an outcome recorded by the end of 2016 due to the duration of TB treatment? Similarly, how many treatment outcomes were recorded in 2019? Would it make more sense to compare 12 month study periods rather than by calendar year since the study period was June to June?
  - *Thank you for the very interesting concern. As suggested, the records considered did not start from the actual year beginning to the end, rather started from nearly the end of the year (in the Ethiopian calendar). As a result, we did not capture the possible number of cases in that same year. Of course, this may be associated with the timing where the facilities started DOTS TB care. In the revised manuscript, we provided the outcomes as suggested. For example, starting in June 2016, 0 % (0/7) had poor TB treatment outcome, 11.2% (16/143) in 2017, the highest was detected in 2018, 16.9 %( 30/177) and then a declining trend of the poor treatment outcome was observed in 2019, 16.4 % (12/73).*
- Analyses: With 58 poor TB treatment outcomes, it appears the adjusted model is at risk for being overfit based on the number of factors (and degrees of freedom) in the adjusted regression model.
  - *Thank you for the suggestion. Considering the reviewers comments and suggestion, in an effort to have adequate sample per variables considered and improve model fitness, we merged variables with several categories (for example occupation), used “age” as continuous which previously entered in the model as categorical, and dropped one variable (disclosure of TB status) in the revised submission. Implementing the recommended changes, positive changes were observed in the strength association and improved precision, confidence interval get narrowed.*
- Discussion: The Discussion is generally underdeveloped. It would be helpful to have a more robust discussion about how the findings of the study can support the local, regional, and national TB program efforts to improve TB treatment outcomes. To that end, reworking the very coarse categorization of Age and distance to health facility as continuous variables may help provide more targeted results for discussion. The Introduction also framed poor treatment outcomes in the context of drug-resistant TB (also noted in the Abstract). However, there is no mention of drug resistance in the Results or in the Discussion.
  - *Thank you very much for the important suggestion. In the revised submission, we have made several improvements based on the reviewers suggestion and improved the discussion. We also edited the introduction to be specific to the problem studied. However, we mentioned in the abstract and introduction section, to show the relation of poor/unsuccessful/ treatment outcome of TB, and the MDR-TB, and to give emphasis about the importance the study on the factors of poor treatment outcome, thereby to the prevention of emerging MDR-TB.*
- **Minor comments**
- Lines 50-51: Would be helpful to update this and include the year so the reader knows when the statistic is relevant. The most recent WHO list of top 10 causes of deaths globally from 2019 no longer includes TB: <https://www.who.int/news-room/fact-sheets/detail/the-top-10-causes-of-death>, though the causes of death in the top 10 vary by country income status according to the WHO.
  - *Thank you, the report was according to 2017 World Health Organization (WHO), so we corrected and indicated the year of report.*
- Line 69: Make sure the treatment outcome category names are used consistently. For example, in line 69 the outcome “defaulter” is listed, but in line 73, “lost to follow-up” is used. If referring to WHO outcomes, the term lost to follow-up is preferred, as noted in the document “Definitions and reporting framework for tuberculosis – 2013 revision (updated December 2014 and January 2020)”.
  - *Thank you very much, we replaced by the term loss to follow-up in the revised submission.*
- Line 69: Where did the successful TB treatment outcomes vary? Was this all of Ethiopia? Please clarify.
  - *Thank you for the clarification question. Yes, it was in Ethiopia. A statement detailing this has been added in the revised manuscript.*
  - Lines 70-75: How do these subgroups relate to the Gibe Woreda noted in the title and which is the site of the study?
    - *Thank you for the comment. Gibe Woreda in one of the Woreda in Southern nation nationalities and people region (SNNPR) of Ethiopia. Regions listed in this part are also the regions of Ethiopia. So all are regions in Ethiopia.*
  - Lines 77-83: this is a long hierarchical list and it is confusing with multiple parentheses that do not all have a closing parenthesis. Please clarify and consider simplifying the text – perhaps with a table.
    - *Thank you for the comment. We corrected in the revised manuscript.*
- Line 88: Please clarify this sentence – what is the comparator for “poorer”? This is the first time in the main text that the authors have written “Gibe Woreda”. Perhaps in the paragraph with lines 69-75 the long list of values could be replaced with context-specific information about Gibe Woreda and how it fits in to Ethiopia and TB program outcomes. Then in the sentence in line 88, clearly identify what you are comparing Gibe Woreda to when you say that treatment “outcome is” (outcomes are?) poorer there.
  - *Thank you for the comment. Our intention here was to explain the status of TB treatment outcomes observed in Gibe Woreda, but the predictors were not studied. Hence, this study investigated the factors of poor TB treatment outcomes.*
- Line 194: what does “about” mean? Is there uncertainty about this number?
  - *Thank you for the comment. In this revised submission, we removed the ambiguity by replacing term previously used by “a total of” 9.3%.*
- Line 196: what does “indifferent enrolment periods” mean?
  - *Thank you for the comment. In this revised submission, we removed the ambiguity by replacing term previously used by “different enrolment periods.”*
- Table 3: what does Disclosure status mean? Earlier in the text and previous table are TB disclosure status and HIV disclosure status, so need to clarify.
  - *Thank you for the comment. It is to refer to “disclosure of TB status”. This has now been corrected in the text and also in Table 3 of the revised submission.*
- Line 232: where are these “other similar settings”?
  - *Thank you for the comment, but we couldn’t find the stated phrase in line 232 of the previous submission.*
- Lines 259-261: This sentence is confusing, please clarify.
  - *Thank you for the comment, and we have clarified it in the revised manuscript.*
- Line 120: ART status mentioned, but not HIV status. Then ART status is not mentioned anywhere else
  - *Thank you for the comment. In revised manuscript, line 126 we corrected and replaced “ART status” by “HIV/AIDS status”. In this study in line 185 and table 2 it was mentioned that “Regarding co-infection with HIV/AIDS, (3.5%) were co-infected with HIV/AIDS”*
- Line 128: smear-negative
  - *Thank you for the comment. In the revised submission, we corrected it as “smear-negative”*
- Line 157: if the statement “it was a good fit…” is a result of the goodness of fit tests, this should be recorded in the Results, not the Methods.
  - *We recorded in method part, b/c it is the result of data analysis part.*
- I would recommend reviewing the manuscript to correct errors in grammar. I would also encourage consistency throughout the manuscript.
- Forexample:
  - In line 56 you should use lowercase ‘t’ for “Mycobacterium tuberculosis” as in line 49.
  - Line 104, 274 uses lowercase ‘w’ for “woreda” but it is capitalized elsewhere.
  - Loss to follow-up is written with and without dashes and abbreviation (LTFU) throughout.
  - Confidence intervals are separated by a comma in some places and dashes in others.
  - *Thank you very much,*
  - *We reviewed the grammar of the manuscript by using freely available software.*
  - *We replaced by “Mycobacterium tuberculosis”, and we made it consistent. We made upper case “Woreda” in all parts of document.*
  - *We have written the “Loss to follow-up” with dashes and the abbreviation form LTF in overall revised document.*
  - *We corrected and separated by dashes in all parts of manuscript.*

Sincerely,

Mathewos Alemu Gebremichael

Department of Public Health,

College of Medicine and Health Sciences,

Arba Minch University, Arba Minch, Ethiopia.

Email: alemumathewos2017@gmail.com
